# Crystal Structure of an Uncommon Cellulosome-Related Protein Module from *Ruminococcus flavefaciens* That Resembles Papain-Like Cysteine Peptidases

**DOI:** 10.1371/journal.pone.0056138

**Published:** 2013-02-14

**Authors:** Maly Levy-Assaraf, Milana Voronov-Goldman, Inna Rozman Grinberg, Gloria Weiserman, Linda J. W. Shimon, Sadanari Jindou, Ilya Borovok, Bryan A. White, Edward A. Bayer, Raphael Lamed, Felix Frolow

**Affiliations:** 1 Department of Molecular Microbiology and Biotechnology, Tel Aviv University, Tel Aviv, Israel; 2 The Daniella Rich Institute for Structural Biology, Tel Aviv University, Tel Aviv, Israel; 3 Department of Chemical Research Support, The Weizmann Institute of Science, Rehovot, Israel; 4 Faculty of Agriculture, Meijo University, Nagoya, Japan; 5 Institute for Genomic Biology, University of Illinois at Urbana-Champaign, Urbana, Illinois, United States of America; 6 Department of Biological Chemistry, The Weizmann Institute of Science, Rehovot, Israel; University of Queensland, Australia

## Abstract

**Background:**

*Ruminococcus flavefaciens* is one of the predominant fiber-degrading bacteria found in the rumen of herbivores. Bioinformatic analysis of the recently sequenced genome indicated that this bacterium produces one of the most intricate cellulosome systems known to date. A distinct ORF, encoding for a multi-modular protein, RflaF_05439, was discovered during mining of the genome sequence. It is composed of two tandem modules of currently undefined function that share 45% identity and a C-terminal X-dockerin modular dyad. Gaining insight into the diversity, architecture and organization of different types of proteins in the cellulosome system is essential for broadening our understanding of a multi-enzyme complex, considered to be one of the most efficient systems for plant cell wall polysaccharide degradation in nature.

**Methodology/Principal Findings:**

Following bioinformatic analysis, the second tandem module of RflaF_05439 was cloned and its selenium-labeled derivative was expressed and crystallized. The crystals belong to space group P2_1_ with unit-cell parameters of *a* = 65.81, *b* = 60.61, *c* = 66.13 Å, β = 107.66° and contain two protein molecules in the asymmetric unit. The crystal structure was determined at 1.38-Å resolution by X-ray diffraction using the single-wavelength anomalous dispersion (SAD) method and was refined to R_factor_ and R_free_ of 0.127 and 0.152 respectively. The protein molecule mainly comprises a β-sheet flanked by short α-helixes, and a globular α-helical domain. The structure was found to be structurally similar to members of the NlpC/P60 superfamily of cysteine peptidases.

**Conclusions/Significance:**

The 3D structure of the second repeat of the RflaF_05439 enabled us to propose a role for the currently undefined function of this protein. Its putative function as a cysteine peptidase is inferred from *in silico* structural homology studies. It is therefore apparent that cellulosomes integrate proteins with other functions in addition to the classic well-defined carbohydrate active enzymes.

## Introduction

Plant cell wall polysaccharides offer an extraordinary source of carbon and energy that can be utilized by various microorganisms, thus contributing a central component to the carbon cycle. In some cases, free-living microorganisms exploit such polysaccharides from decaying plants; in other cases symbiotic microbes assist both invertebrates (e.g., termites) and higher vertebrates (e.g., ruminants) in converting plant-derived polysaccharides to digestible components. Cellulose is the main structural component of the plant cell wall. It is arranged in highly recalcitrant fibrils which are usually embedded in a colloidal matrix of hemicellulose and lignin [1].

The cellulolytic, fiber-degrading bacterium *Ruminococcus flavefaciens* is one of the critically important inhabitants in the rumen of herbivores, which plays a central role in the degradation of plant cell wall fiber. *R. flavefaciens* FD-1 is a gram-positive cellulosome-producing anaerobic bacterium [2]–[6] that possesses the most elaborate cellulosome system thus far discovered in nature. Over 220 different dockerin-containing open reading frames (ORFs) have been identified in this strain [7], [8], which is more than triple the number so far detected in the original model bacterium, *Clostridium thermocellum*, from which the cellulosome concept was first described. Only 36% of these ORFs were predicted to be carbohydrate-acting enzymes. Other ORFs were bioinformatically predicted as structural and catalytic protein modules, such as proteases (peptidases), transglutaminases, lipases and leucine-rich repeats (LRRs) [9]. These components have not been known previously to be associated with polysaccharide degradation, and their presence as part of the cellulosome system is particularly intriguing.

**Figure 1:**
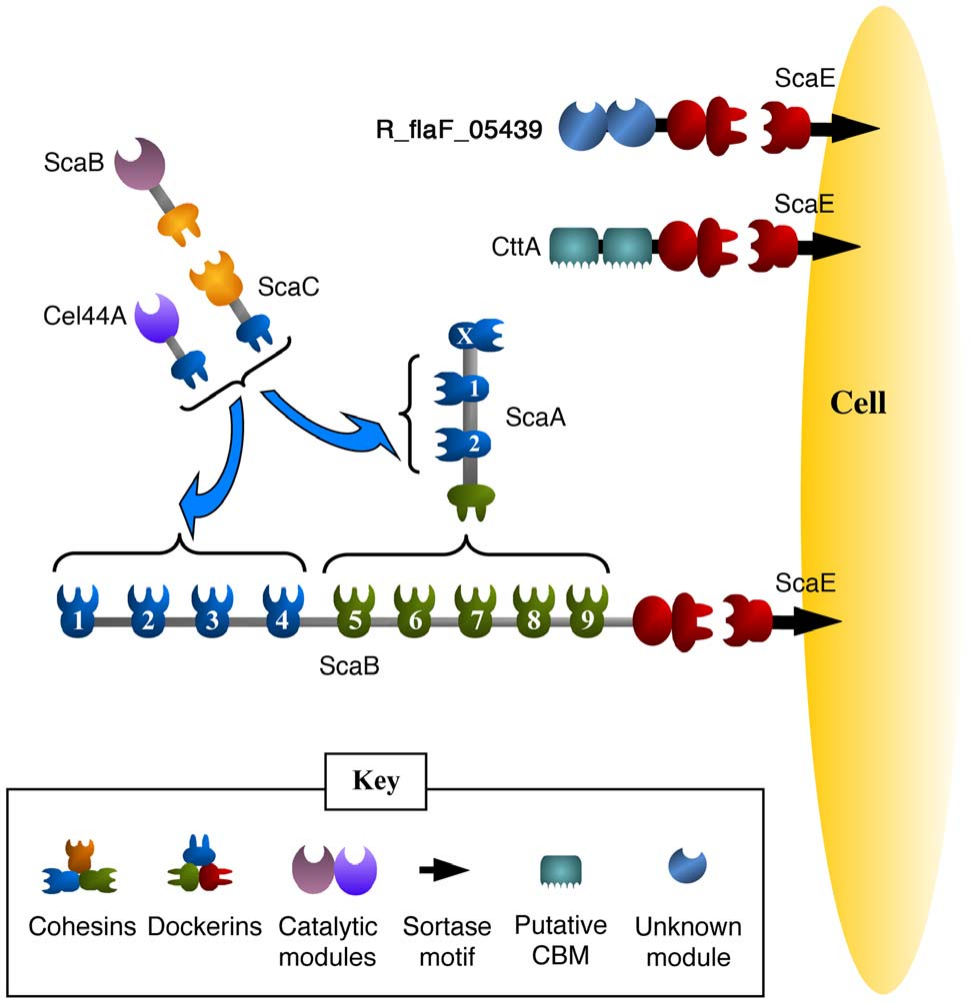
Schematic model of the proposed arrangement of the *R. flavefaciens* FD-1 cellulosomal components. The components of the cellulosome include the various scaffoldins (ScaA, ScaB, ScaC and ScaE) and the dockerin-containing catalytic subunits (represented in the figure by the Ce3B-type and Cel44A-type). ScaE is implanted into the cell surface via a sortase-like signal motif. Like the cellulosomal ScaB, two other cellulosome-related proteins, CttA and RflaF_05439, contain an XDoc and are also attached to the cell surface via the ScaE-Coh. The *R. flavefaciens* FD-1 cellulosome is organized as follows: The ScaA cohesins bind either to the ScaC dockerin or to the various Cel44A-type dockerins, and the lone ScaC cohesin serves as an “adaptor scaffoldin”, which selectively binds to the Ce3B-type dockerins and incorporates their parent proteins into the complex. ScaA is attached via its dockerin into ScaB cohesins 5–9, whereas the specificity of ScaB cohesins 1–4 is similar to those of ScaA.

In *R. flavefaciens*, the key cellulosomal scaffoldin components (ScaA, ScaB, ScaC, CttA and ScaE) are organized into a *sca* gene cluster whose presence has been documented in five different strains of this species [10]. The complement of interconnecting cellulosomal components is anchored to the cell surface via interaction of the X-dockerin (XDoc) modular dyad of the ScaB subunit with the cohesin (Coh) of ScaE [11], which is covalently implanted into the cell surface via a sortase-like signal motif (Figure 1). The ScaE-Coh also binds to the same type of XDoc module present on another cellulosome-related protein, CttA. The modular dyad of the CttA homologue from *R. flavefaciens* 17 was demonstrated to bind to the cellulose substrate [12].

**Figure 2:**
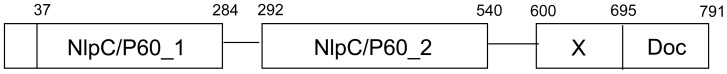
Schematic representation of the modular structure of RflaF_05439. The protein contains a signal peptide followed by two modules, predicted to belong to the NlpC/P60 superfamily (NlpC/P60_ 1 and NlpC/P60_ 2) and an X-dockerin (XDoc) modular dyad.

A third XDoc modular dyad was identified by bioinformatics data mining of the *R. flavefaciens* FD-1 genome. The parent protein, RflaF_05439 (accession number: ZP_06142651), consists of a tandem repeat of two conserved modules (Figure 2) at the N terminus and a C-terminal XDoc, which also binds to ScaE-Coh (unpublished results). Protein BLAST [13] searches of the NCBI database using either of the latter repeated segments failed to reveal similar sequences in other species, and functional information was not evident prior to determination of the structure described in this work. In the present communication, we present the crystal structure of the second tandem module of this protein. *In silico* analysis of the structure implied that the protein belongs to an established superfamily of cysteine-like peptidases, termed NlpC/P60, and this domain will hereafter be coined NlpC/P60_2.

## Experimental Methods

### Cloning, Expression and Purification

The DNA encoding NlpC/P60_2, the second tandem module of the parent protein RflaF_05439, was amplified from genomic DNA of *R. flavefaciens* FD-1 using F- CGGCTAGCATGTACAATTCCGACGGCTGGTACA and R- CCGCTCGAGTTACGTTACCACAGGTTCTGCTTTCT primers. The insert was cloned into the pET28a expression vector (Novagen) with a sequence encoding for a hexa-His tag attached to the 5′ end, using *Nde*I and *Xho*I restriction enzymes. The construct was expressed in *Escherichia coli* strain BL21(DE3) RIL. Expression of the seleno-l-methionine-labelled NlpC/P60_2 was conducted according to the method described previously [14]. The expressed His-tagged protein was purified by metal-chelate affinity chromatography using a Ni-IDA resin (Rimon Biotech, Israel) with phosphate buffer (50 mM NaH_2_PO_4_, pH 6.0, 300 mM NaCl and 10% glycerol). Fast protein liquid chromatography (FPLC) using an AKTA-prime system (Amersham Pharmacia Biotech, Piscataway, NJ) was performed on a Superdex^®^75 XK 16 (Pharmacia Biotech) pre-packed column in column buffer (50 mM Tris pH 7.5, 0.15 M NaCl, 0.05% sodium azide). The protein was concentrated using Centriprep YM-3 centrifugal filter devices (Amicon Bioseparation, Millipore, Inc., Billerica), yielding 1.5 ml of purified concentrated protein (20 mg ml^−1^). Protein concentration was determined by measuring the absorbance at a wavelength of 280 nm using the calculated extinction coefficient of the protein (ε_280_ [g/l] = 0.92).

### Crystallization, Data Collection and Processing

Crystallization was performed using a Hampton Research Index-H™ kit, employing the microbatch crystallization method under 1∶1 mixture of silicon and paraffin oil [15] at 293 K. Samples were dispensed using an Oryx 6 Crystallization Robot from Douglas Instruments (http://www.douglas.co.uk/). Initial SeMet NlpC/P60_2 multi-layered crystals were obtained after 6 days in a 2-µl drop containing 1-µl protein solution and 1 µl of reservoir solution consisting of 0.2 M ammonium acetate, 0.1 M BIS-TRIS pH 6.5 and 25% w/v polyethylene glycol 3350. Further optimization experiments were performed using the hanging-drop method in a 4-µl drop consisting of 2-µl protein solution and 2-µl reservoir solution equilibrated against 0.4-ml reservoir solution. The streak seeding technique [16] with an eyelash (Ted Pella, Inc; www.tedpella.com) was employed in order to improve clustered crystals. Crystals from the final reservoir solution consist of 0.2 M ammonium acetate, 0.1 M BIS-TRIS pH 6.5 and 23% (*w/v*) polyethylene glycol 3350. Crystals were harvested from the crystallization drop using a MiTeGen MicroMount (http://www.mitegen.com) made of polyimide and transferred for a short time into a cryo-stabilization solution mimicking the mother liquor supplemented with 18% (*w/v)* sucrose, 16% (*w/v*) glycerol, 16% (*w/v*) ethylene-glycol and 4% (*w/v*) glucose. For data collection, crystals were mounted on the MiTeGen MicroMount and flash-cooled in a nitrogen stream at a temperature of 100 K produced by an Oxford Cryostream low-temperature generator [17]. Single-wavelength Se-SAD diffraction data were measured to a resolution of 1.38 Å with wavelength tuned to 0.9795 Å to enhance the anomalous signal from the Se atoms. Oscillation range of 0.5° per frame over 360° rotation was implemented. Data were indexed, integrated and scaled using DENZO and SCALEPACK as implemented in HKL2000 [18]. During scaling, Friedel pairs of reflections were kept separate for subsequent use. Details of the data-collection statistics are given in Table 1.

**Table 1:** Data-collection, processing and refinement statistics for crystal of SeMet derivative of NlpC/P60_2.

Experimentalconditions	
X-ray source	ESRF ID23-1
Wavelength (Å)	0.9796
Temperature (K)	100
Detector	CCD ADSC
**Crystal parameters**	
Space group	P2_1_
Unit cell parameters (Å and °):	
a	65.81
b	60.61
c	66.13
β	107.66
Resolution (Å)	32.4–1.38 (1.40–1.38)
**Data processing**	
No. of reflections	715274
No. of unique reflections #	182049
Completeness (%)	95.9 (83.5)
Mean I/σ(I)	37.5 (3.5)
R_merge_ ##	0.051 (0.449)
Redundancy	7.3(5.6)
**Structure refinement**	
Resolution range (Å)	32.39–1.38
R_work_	0.127 (0.1567)
R_free_	0.152 (0.1782)
No. of reflections in test set ###	3825
No. of reflections used in refinement ###	178224
**Average B factor (Å^2^)**	
Overall	18.3
Atoms in protein	14.4
Atoms of ligands	39.1
Atoms of solvent	20.9
R.M.S. deviation, bond lengths (Å)	0.009
R.M.S. deviation, bond angles (°)	1.203
**Ramachandran statistics**	
Permitted (%)	2.9
Favored (%)	97.1

Values for the highest resolution shell are given in parentheses.

# *R*_mearged_ = Σ*_hkl_* Σ*_i_|I_i_(hkl) – <I(hkl)>|/*Σ*_hkl_* Σ*_i_I_i_*(*hkl*), where Σ*_hkl_* denotes the sum over all reflections and Σ*_i_* the sum over all equivalent and symmetry related reflections [44].

## Friedel pairs of reflections were not merged to preserve the anomalous signal.

### For the participation in the test set both reflections comprising a Friedel pair were selected.

### Structure Determination and Refinement

The phases of the structure were determined using *SHELXC/D/E* [19] as employed in the HKL2MAP [20] graphical user interface. Heavy atom substructure containing all ten possible Se-atom sites was identified by *SHELEXD*; primary phasing, phase modification and initial Cα tracing were obtained using *SHELXE.* After density modification by DM [21], auto model building was implemented in Arp/WARP [22]. The final structure was refined using PHENIX [23] to a crystallographic R_work_ and R_free_ of 12.91% and 15.32%, respectively. During refinement, Friedel pairs of reflections were kept separated, anomalous dispersion parameters f’ and f” were not refined, and manual corrections and structure validation were performed using *COOT* [24]. A total of 494 amino acid residues and 722 water molecules were present in the final model. Coordinates and structure factor amplitudes for NlpC/P60_2 have been deposited in the PDB (accession code 4EYZ).

## Results and Discussion

### Overall Structure of the RflaF_05439 Second Repeat

**Figure 3:**
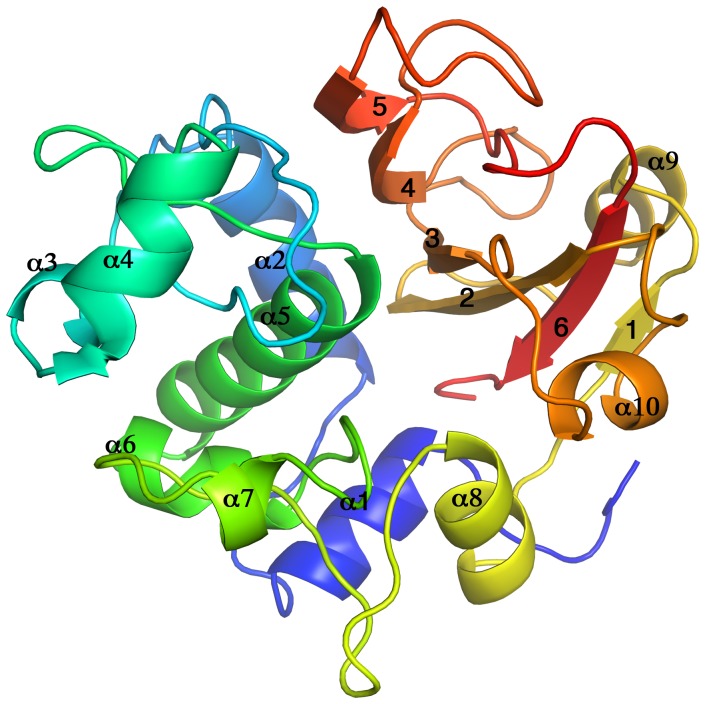
Cartoon diagram of the overall three-dimensional structure of NlpC/P60_2. Rainbow-colored α-helixes and β-strands are shown from blue at the N-terminus to red at the C-terminus. The β-sheet (formed by antiparallel β-strands 1-6-2-3-4-5) is flanked by short α-helixes (3-4-7-9) and a globular α-helical domain (formed by α-helixes 1-2-5-6). All 10 α-helixes are located at the N terminus, followed by the six β-strands.

The X-ray crystal structure of the second module of the tandem repeat of RflaF_05439 from *R. flavefaciens* FD-1 was determined at a resolution of 1.38 Å. There are no significant differences between the two molecules in the asymmetric unit (RMSD of 0.06 Å for all Cα atoms). The N-terminal His tag was not modeled into the structure owing to the weak electron density. The overall fold consists of two globular domains that form a tight interface: an N-terminal α-helical domain and a C-terminal domain comprised of 6 antiparallel βstrands covered by short α-helices and loops (Figure 3).

### Detection of the NlpC/P60 Superfamily Signature

In an attempt to elucidate a biological function for RflaF_05439, structural homology searches of the RCSB Protein Data Bank queried with the structure of the second module of the tandem repeat were performed using the DALI search engine [25]. Notable similarity was observed to a putative “xylanase” from *Bacteriodes fragilis* (PDB entry 2P1G, Z score of 7.8, unpublished) and to a putative endopeptidase from *Anabaena variabilis* (PDB entry 2HBW, Z score of 7.5, [26]) that belong to the NlpC/P60 superfamily.

NlpC/P60 is an example of a superfamily with members sharing structural similarities that cannot be detected at the sequence level [27]. The NlpC/P60 superfamily of peptidases is characterized by a conserved catalytic triad, which contains cysteine, histidine and a polar residue (i.e., glutamine, aspargine, histidine, glutamate or aspartate) within a catalytic core. Beyond the catalytic core, the structures are highly divergent.

Identification of the second module of the tandem repeat as a candidate member of the NlpC/P60 superfamily according to its 3D structure facilitated further analysis of sequence homology. Interestingly, we found and sequenced a homologous RflaF_05439 gene from the closely related *R. flavefaciens* strain 17. It should be noted that although the two strains (FD-1 and 17) belong to the same species, their cellulosomal organization is, in some of their respective components, dissimilar [10], [28]. The homologous gene shows a similar organization of two repeated modules separated by a linker sequence and a C–terminal XDoc. RflaF_05439 of *R. flavefaciens* FD-1, and its homolog of strain 17 shares overall sequence similarity of 39%, with increased similarity of 71% between the respective second NlpC/P60 repeats.

**Figure 4:**
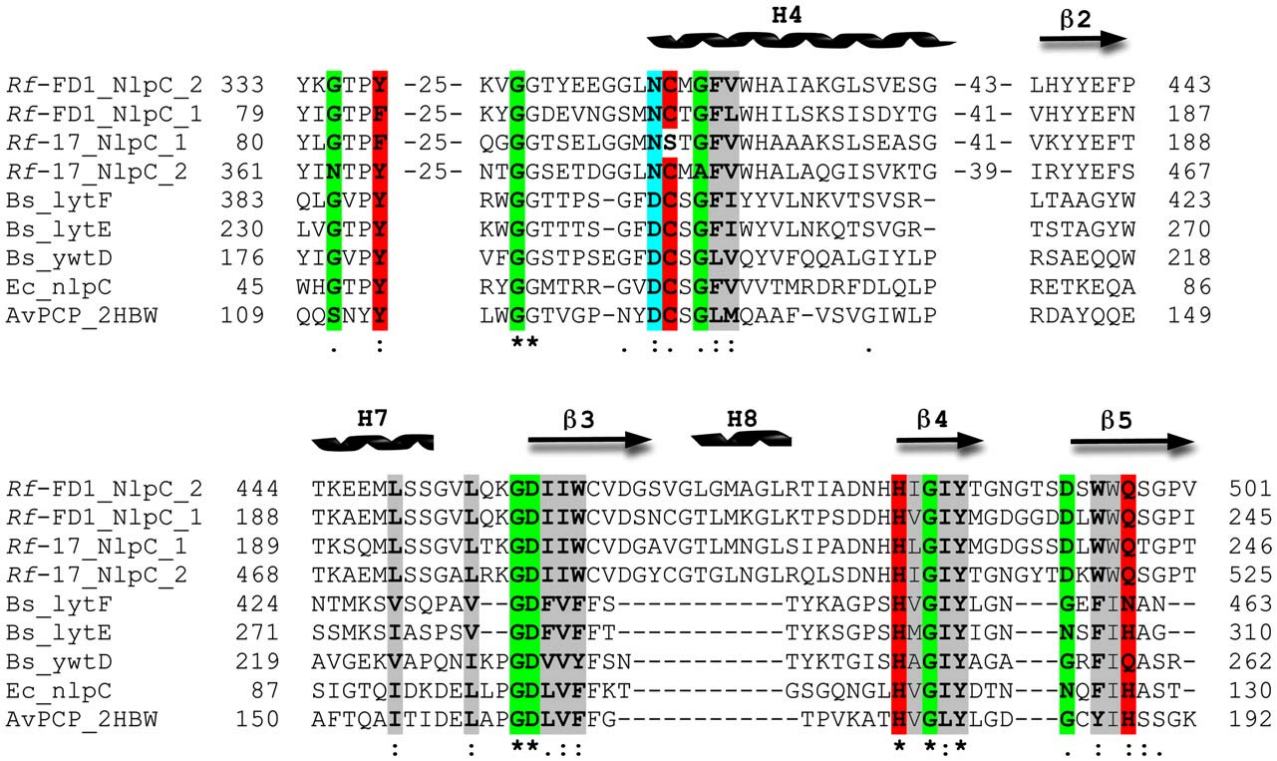
Multiple sequence alignment of selected portions of the RflaF_05439 NlpC/P60 repeats from *R. flavefaciens* strain FD-1 with those of the homologous protein in strain 17 and selected members of the NlpC/P60 superfamily. Multiple sequence alignments were constructed using ClustalW. The numbers within the alignment represent corresponding stretches of non-conserved residues that are not shown. Highly conserved residues in the NlpC/P60 superfamily are highlighted as follows: red – catalytic (including Tyr which is responsible for oxyanion hole formation), gray - hydrophobic, green - small, cyan - polar. Symbols: asterisks - full conservation; colons - strongly similar; periods - weakly similar. The sequences are denoted by their gene name preceded by the species abbreviation: Rf, *R. flavefaciens*; Bs, *Bacillus subtilis*; Ec, *Escherichia coli*. AvPCP represents endopeptidase from *Anabaena variabilis* denoted by its PDB code (2HBW). Secondary structural elements (enumerated β strands and α helixes) are indicated.

Multiple sequence alignment of the repeated NlpC/P60 modules of RflaF_05439 from the two strains and five other members of the NlpC/P60 superfamily was performed. The alignment emphasizes the conservation of the catalytic triad (Figure 4). The catalytic histidine residue [29] is strictly conserved among all the NlpC/P60 superfamily members presented in the figure, whereas the catalytic polar residue is divergent. The catalytic cysteine is highly conserved with the notable exception of the first repeat of the homologous module from *R. flavefaciens* 17. In the latter module, the conserved catalytic cysteine is substituted by serine. The appearance of a catalytic serine, a catalytic histidine and a catalytic polar residue characterizes serine proteases and lipases [27]. Other elements characterizing the NlpC/P60 superfamily, such as specific polar residues, glycines, hydrophobic residues, and small amino acids [27], are also highly conserved in the RflaF_05439 modules. Owing to this analysis, the two tandem repeated modules were hereafter referred to as NlpC/P60_1 and NlpC/P60_2, respectively.

### Structural Homology with the NlpC/P60 Superfamily

**Figure 5:**
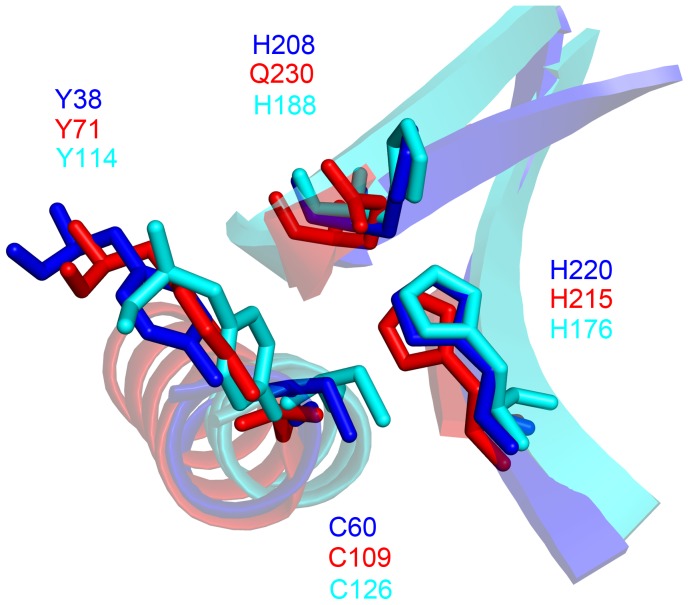
Structural superposition of the catalytic triad of members of the NlpC/P60 superfamily. The catalytic triad of NlpC/P60_ 2 (blue, PDB code 4EZY), the putative “xylanase” (red, PDB code 2P1G), and AvPCP (cyan, PDB code 2HBW). The fourth functionally important tyrosine residues in NlpC/P60_2 (Tyr71), 2P1G (Tyr38) and 2HBW (Tyr114) are also shown.

Structural superposition of the putative catalytic triads of NlpC/P60_2 [30], [31] and two other NlpC/P60 superfamily members – a putative “xylanase” from *Bacteriodes fragilis* (PDB code 2P1G, unpublished) and AvPCP, a peptidoglycan cysteine endopeptidase from *Anaebena variabilis* (PDB code 2HBW, [26]) – is presented in Figure 5. The spatial configuration of all catalytic residues (Cys, His and Gln/His/Asn) is strictly conserved, with RMSD values ranging from 0.04 to 0.14 Å. A fourth residue, Tyr71, is also conserved. This residue is important for formation of a putative oxyanion hole, which helps stabilize transitional forms between the peptidase and the substrate [30]. Beyond the catalytic core, the structures are highly divergent through deletions and insertions of structural elements. We therefore suggest that the same structural arrangement of the catalytic residues in NlpC/P60_2 serves to classify the module in the NlpC/P60 superfamily.

**Figure 6:**
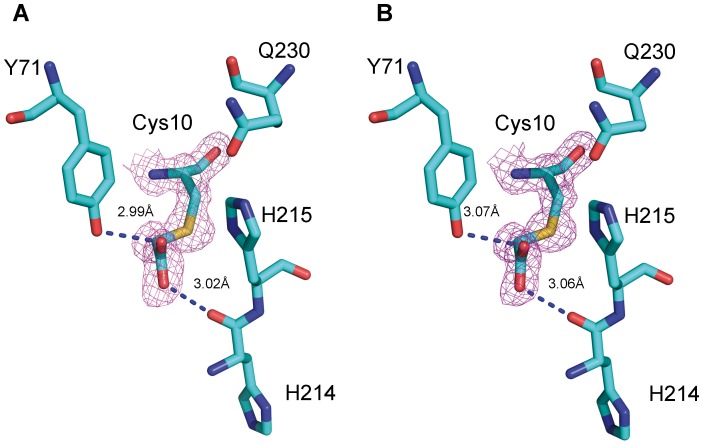
View of the modified active-site cysteine residue shown in both subunits. A.) Subunit A. B.) Subunit B. The 2Fobs – Fcalc electron density map, displayed around the cysteine amino acid residue and rendered at 1.2 RMSD level, is shown in magenta. Various moieties may fit the density map (see text for details), the best of which is an acetate group.

In the NlpC/P60_2 structure, extra electron density was discovered contiguous with the side chain of the catalytic cysteine, Cys109 (Figure 6). This extraneous electron density was modeled as acetate. Both oxygen atoms of acetate fit well the electron density map and form hydrogen bonds with neighboring residues and water. The fact that we could not detect protease activity in the purified protein, coupled with the observed derivatization of the presumed active-site cysteine, would suggest that the modification originated during expression and/or purification, perhaps producing a suicide complex during enzymatic reaction. Further experiments will be necessary in order to determine the precise nature of this modification. Various alternatives for the observed electron density can also be considered, including ethanethioamide, acetone or acetamide. In any case, is it likely that the modified cysteine would lead to an inactivated enzyme, which may explain why attempts to demonstrate protease activity were unsuccessful.

### Active Site Groove

**Figure 7:**
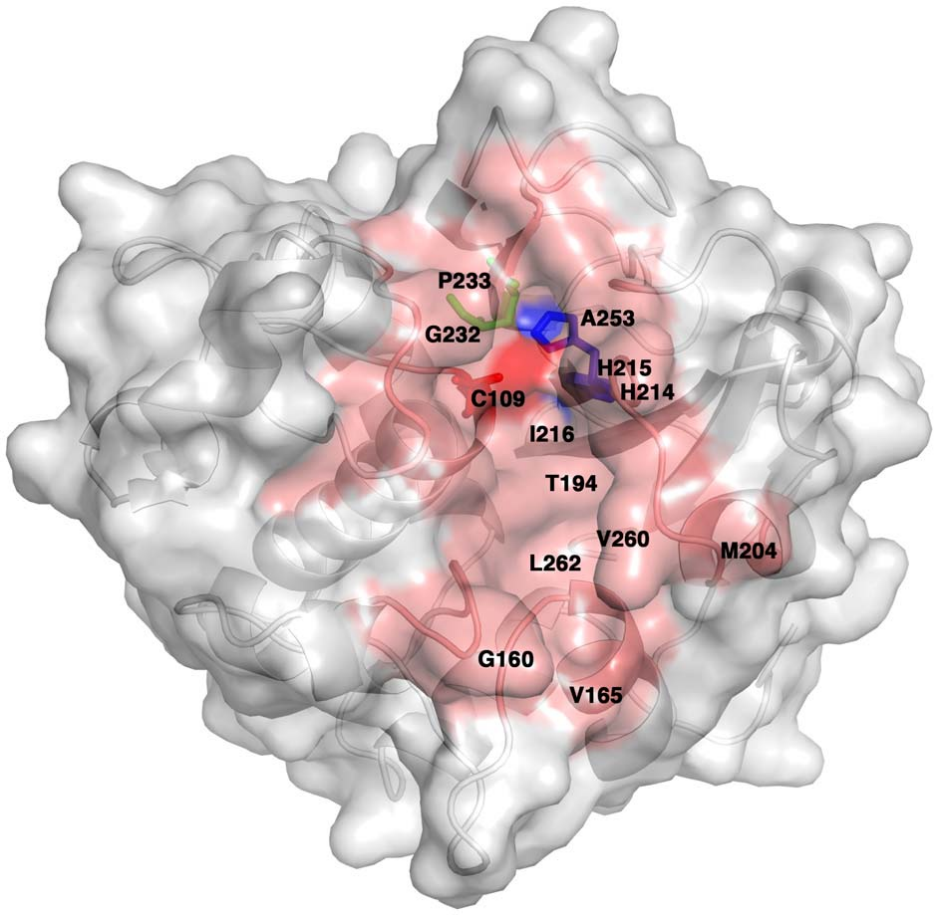
Putative active-site groove of NlpC/P60_2. The residues that form the groove are labeled on the surface representation of the molecule. The cysteine residue (C109) in the catalytic triad is colored red, and the histidine (H215) is colored blue. As in the other known NlpC/P60 structures, the glutamine residue (Q230) is buried. Residues that form the active site are colored salmon red, and indicated by single amino acid code and number.

The potential active site of NlpC/P60_2 is located in the channel groove that runs along the interface between the N-and C-terminal domains (Figure 7). The entrance to the groove is formed by the following amino acid residues: Asp73, Tyr74, Val75, Asn108, Phe138, Gly159, Thr162, Val163, Ser139, Ser140, Tyr141, Arg207, Ile210, Ala211, Asp212, Asn213 and Leu240. The middle section of the groove consists of Tyr71, Met110, Pro137, Trp161, Thr209 and Leu207. Residues lining the bottom of the groove are Cys109, Gly160, Val165, Trp194, Met204, His214, His215, Ile216, Gly232, Pro233, Ala253, Val260 and Leu262.

**Figure 8:**
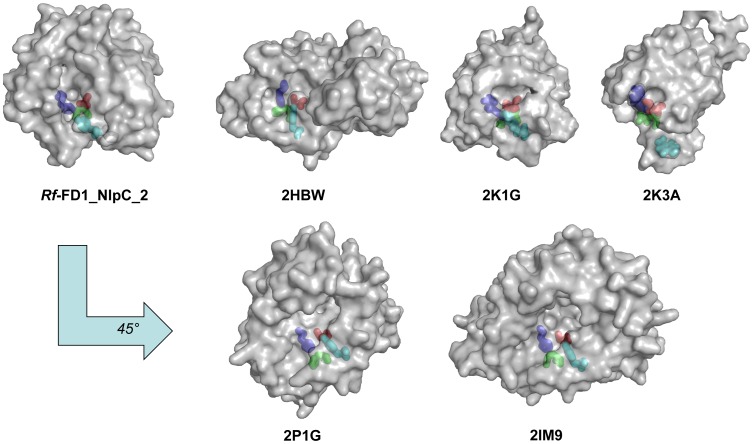
Location of the active-site grooves of various members of the NlpC/P60 superfamily represented on the molecular surface (annotated by PDB entry number). The catalytic triad residues are colored as follows: cysteine in red, histidine in blue, and the third polar residue in green. The active-site grooves of 2HBW, 2K1G, 2K3A are located on the same face as that of NlpC/P60_2. The active-site residues of all structures were first superimposed; in order to view the grooves of 2P1G and 2IM9. The latter structures were then rotated approximately 45° counter-clockwise, relative to NlpC/P60_2.

Compared to several other proteins from this superfamily whose structures have been determined (PDB codes 2HBW [26], 2K1G [32] and 2K3A [33]), the relative position of the active-site groove of NlpC/P60_2 is located approximately on the same face of the molecule (Figure 8). In contrast, in the structures of two other proteins (2P1G and 2IM9, both unpublished), the relative orientation of the active-site grooves is rotated approximately 45° about the X axis with respect to NlpC/P60_2.

### Potential Role of RflaF_05439 in the Cellulosome System of *R. flavefaciens*

Information concerning the various cellulosomal proteins and, in particular, uncommon components that are not of the classic cellulosome mold is necessary in order to extend the cellulosome paradigm of protein complex assembly beyond the realm of glycoside hydrolases and other carbohydrate-active enzymes. This has been enabled in recent years by extensive genome-sequencing efforts and the gradual accumulation of genome sequences of cellulosome-producing bacteria, thus providing a genome-wide view of the cellulosome components and related proteins – a recently emerging field termed cellulosomics [1]. For example, the sequenced genome of the prominent anaerobic, thermophilic cellulosome-producing bacterium, *Clostridium thermocellum*, revealed the presence of genes encoding dockerin-containing serine proteinase inhibitors (serpins) [34] and a subtilisin-like serine protease [35]. Nevertheless, the great majority of the >70 dockerin-containing proteins in the *C. thermocellum* cellulosome are indeed carbohydrate-active enzymes.

In contrast to *C. thermocellum*, the cellulosomal system in *R. flavefaciens* includes an abundance of over 220 different dockerin-containing proteins, only about half of which can be classified as carbohydrate-active enzymes. The remainder includes putative peptidases, serpins, structural proteins, and proteins of unidentified function [9].

The crystal structure of the protein module described in this work allows the assignment of a putative function for the RflaF_05439 protein, which is identified to be a member of the NlpC/P60 superfamily, possessing the characteristic conserved catalytic triad residues (Cys109/His215/Gln230). Interestingly, the association of this protein module with this superfamily of peptidases was not evident from the primary sequence, but could only be determined from its structural characteristics. All functionally approved members of the NlpC/P60 family are γ-glutamyl D,L-endopeptidases. They were detected in *Bacillus subtilis* [36]–[39], *Streptococcus mitis* [40], *Streptococcus aureus* [41] and in bacteriophage [2]. The same structural arrangement of the catalytic core of the NlpC/P60 superfamily suggests that the mechanism of proteolysis is expected to be similar. However, at this point, definitive proof is still wanting, and it can only be speculated that RflaF_05439 functions as a cysteine peptidase.

Interestingly, NlpC/P60 proteins are usually characterized by a single catalytic NlpC/P60 domain. One exception is YwtD from *B. subtilis* that carries 3 copies of this domain while only the second repeat is functional [38]. RflaF_05439 is the only current example of a duplicated NlpC/P60 domain [42].

The dockerin module of RflaF_05439 interacts with ScaE-Coh (unpublished results), and ScaE-Coh is attached covalently to the cell surface via a sortase-like signal motif [12], [43]. Its localization on the cell surface would support involvement in interactions with the environment, and RflaF_05439 may have a novel type of functionality that remains to be determined. In this context, it might play a role in supporting cell-cell interactions or dynamics or might have other unknown regulatory roles, serving, for example, as a protease that protects the bacterium from environmental hazards. Further studies are necessary in order to understand the function of this cellulosome-related protein from *R. flavefaciens* FD-1.
